# Transmission Dynamics of Gilts Persistently Infected with Atypical Porcine Pestivirus

**DOI:** 10.3390/v18060590

**Published:** 2026-05-23

**Authors:** Alexandra C. Buckley, Bailey L. Arruda, Juan-Carlos Mora-Díaz, Ronaldo L. Magtoto, Luis G. Gimemez-Lirola, Shollie M. Falkenberg

**Affiliations:** 1 Virus and Prion Research Unit, National Animal Disease Center, Agriculture Research Service, U.S. Department of Agriculture, Ames, IA 50010, USA; 2 Department of Veterinary Diagnostic and Production Animal Medicine, Iowa State University, Ames, IA 50011, USA; 3 Ruminant Disease and Immunology Research Unit, National Animal Disease Center, Agricultural Research Service, U.S. Department of Agriculture, Ames, IA 50010, USA

**Keywords:** atypical porcine pestivirus, APPV, congenital tremor, longitudinal study, transmission

## Abstract

Atypical porcine pestivirus (APPV) is a pestivirus that infects swine and has been associated with congenital tremor (CT) in piglets in the field, as well as those born to experimentally infected sows. However, APPV has also been detected in swine of various ages without clinical signs. Experimental and field studies have demonstrated prolonged detection of viral RNA in serum, secretions, and tissues. For this work, nine gilts from a longitudinal APPV field study were selected based on birth in CT-positive litters and evidence of prolonged detection of virus in the sera. These six-month old animals were transported to a research facility for further sampling, breeding, and necropsy. The gilts were placed in contact with naïve animals for approximately one month at two different timepoints, prior to and during gestation, to assess transmission. After farrowing, litters were monitored for CT and tested for APPV. Following arrival, serum samples were PCR-negative for APPV; however, the gilts consistently tested positive in oral fluids and had detectable APPV in the cerebellum months later at necropsy. The gilts had a delayed humoral immune response, with the majority not seroconverting until approximately ten months of age. There were PCR-positive tissues and evidence of seroconversion in animals from both contact groups; however, none of the litters tested PCR-positive for APPV. These findings improve our understanding of the temporal dynamics and transmission potential of APPV infection and can help guide control measures to reduce viral spread.

## 1. Introduction

Atypical porcine pestivirus (APPV), also known as *Pestivirus scrofae*, is a single-stranded, positive-sense RNA virus in the genus *Pestivirus* of the family *Flaviviridae*. Sequencing efforts led to the discovery of APPV, and its association with congenital tremor (CT) in piglets, a condition observed in the swine industry for over one hundred years [1,2]. Congenital tremor in swine has multiple etiologies, including genetic defects, toxicosis, and viral infections such as classical swine fever virus (CSFV) [3]. Extensive testing of swine samples identified APPV in animals of all ages, not only in piglets exhibiting CT [4,5]. Subsequently, the virus has been detected globally in both domestic and wild pig populations [6,7,8,9,10,11,12]. Similar to other pestiviruses, APPV exhibits substantial genetic diversity, even within the same geographical area [4,13,14].

Longitudinal sampling demonstrated that APPV RNA can be detected in serum, feces, and oral fluids for an extended period, and infected animals may remain asymptomatic [15,16,17,18]. In some animals, delayed or absent antibody responses have been reported, which could play a role in the prolonged viral detection [17,19]. Pestiviruses, especially those infecting ruminants like bovine viral diarrhea virus and border disease virus, can result in persistently infected (PI) animals depending on the timing of infection during gestation [20]. These PI animals are immunotolerant and recognize the virus as self [21]. In swine, CSFV has also been shown to cause persistent infection and immunosuppression when infection occurs postnatally [22]. Isolation and propagation of APPV in cell culture have historically been difficult, limiting further experimental characterization. Consequently, it has been difficult to assess whether PCR-positive field samples contain infectious viruses, especially samples with low viral loads. To gain a better understanding of both the horizontal and vertical transmission potential of animals that persistently test PCR-positive for APPV, nine gilts previously enrolled in a longitudinal field study were selected at approximately six months of age for further sampling and breeding at a research facility. These gilts were born in CT-positive litters and consistently tested PCR-positive for APPV in the sera [19]. The objectives of this study were to characterize: (1) the duration of viremia and antibody response in the gilts born in CT/APPV-positive litters, (2) the transmission potential of animals persistently APPV PCR-positive over time, and (3) whether these gilts produce APPV- and CT-positive piglets.

## 2. Materials and Methods

All procedures performed on animals in this study were approved by the National Animal Disease Center (NADC) institutional animal care and use committee. Animals were euthanized by intravenous administration of a barbiturate (Fatal Plus, Vortech Pharmaceuticals, Dearborn, MI, USA).

Nine gilts were delivered to the NADC in Ames, IA, from a commercial swine farm in the Midwestern United States. Pigs from this farm had previously been enrolled in a longitudinal study to track APPV infection dynamics and humoral immune response following an outbreak of CT [19]. These gilts were selected based on birth in CT-positive litters and persistent APPV PCR-positive serum during the previous study. All gilts except one, gilt 54, were observed with CT. After arrival at the NADC, the gilts were sampled monthly (blood, oral swabs, and rectal swabs), and group oral fluids were collected to monitor APPV by nucleic acid detection and antibody response.

**Figure 1:**
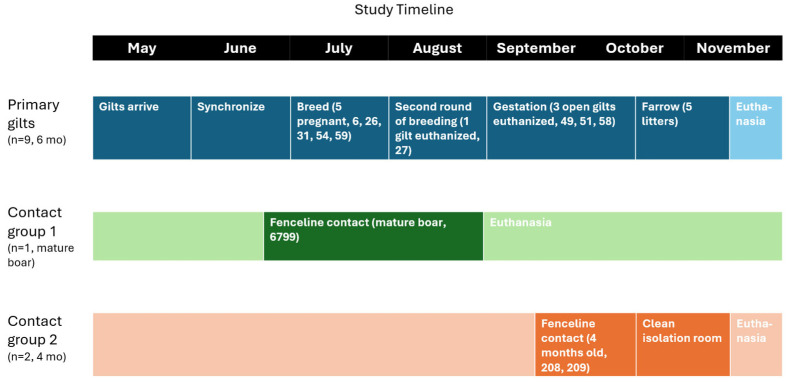
Timeline detailing arrival, breeding, and farrowing of the primary gilts (blue). In addition, the timing of contact placement for contact group 1 (green) and contact group 2 (orange) is shown. Darker colors represent times when the animals were actively involved in the study.

The study timeline is depicted in Figure 1. One gilt (27) did not reach an adequate size for breeding and was euthanized for tissue collection. The remaining eight gilts were synchronized and bred via artificial insemination with semen confirmed PCR-negative for APPV. A boar (6799, contact group 1) was placed in fenceline contact for heat detection during artificial insemination and remained in contact for approximately two months prior to euthanasia. Three gilts (49, 51, 58) did not conceive and were euthanized for tissue collection. During the last third of gestation, two four-month-old pigs (208, 209, contact group 2), one male and one female, were placed in fenceline contact with the five pregnant gilts (6, 26, 31, 54, 59) for one month. Contact group 2 animals were sampled weekly (blood, oral swabs, and rectal swabs) during the contact period and for an additional month after removal from the gilts while housed in a separate animal space.

To assess vertical transmission, the gilts and their litters were sampled (blood and oral swabs) on the day of farrowing. Litters were observed daily for clinical signs of CT, classified as present or absent based on visible tremors. At the end of the study (~35 days after farrowing), all the gilts and piglets were sampled and euthanized. Tissues were collected from two piglets from each litter.

Blood samples were collected from the jugular furrow into serum separator tubes (BD Vacutainer^®^, Franklin Lakes, NJ, USA), and serum was aliquoted and stored at −80 °C until testing. Swabs were collected in 2 mL of minimum essential medium (MEM) and oral fluids were collected with cotton ropes and recovered into 50 mL conical tubes [23]. All samples were stored at −80 °C until testing. Tissues collected at necropsy included cerebellum, tracheobronchial lymph node (TBLN), tonsil, mandibular salivary gland, palatoglossal arch, and mandibular lymph node.

Real-time reverse transcription PCR (RT-qPCR) was performed as previously described [19]. Briefly, tissues were thawed and resuspended in TRI-Reagent^®^ (Life Technologies, Carlsbad, CA, USA) in gentleMACS™ M tubes (Miltenyi Biotec, Bergisch Gladbach, Germany). Tissues were dissociated using a gentleMACS™ Octo-Dissociator (Miltenyi Biotec). The MagMAX™-96 for Microarrays Total RNA Isolation Kit (Applied Biosystems, Waltham, MA, USA) was used for tissue samples, while the MagMAX™ Pathogen RNA/DNA kit (Applied Biosystems) was used for all other samples following the manufacturer’s instructions. Both kits were run on a MagMAX™ Express Magnetic Particle Processor (Applied Biosystems). Next, 5 µL of extracted product was added to 20 µL of the AgPath-ID™ One step RT-PCR master mix (Applied Biosystems) and reactions were performed on an ABI 7500 Fast instrument (Applied Biosystems). Ct values greater than 35 were classified as negative.

Serum samples were tested for antibodies against the E^rns^ protein using an indirect ELISA, as described previously [24]. In brief, 96-well polystyrene ELISA plates (Thermo Fisher Scientific, Waltham, MA, USA) were coated with 100 μL of diluted APPV E^rns^ (0.33 μg/mL). Coated plates were loaded with 100 μL/well serum diluted at a ratio of 1:100 in phosphate-buffered saline (PBS) containing fetal bovine serum (FBS; Gibco^®^, Thermo Fisher Scientific), and incubated at 37 °C for 1 h. Plates were washed, followed by the addition of 1:25,000 peroxidase (HRP)-conjugated goat anti-pig IgG antibody (Bethyl Laboratories Inc., Montgomery, TX, USA) diluted at 1:25,000 per well, and incubated at 37 °C for 1 h. After another washing step, tetramethylbenzidine–hydrogen peroxide (TMB) substrate solution (Surmodics IVD, Inc., Eden Prairie, MN, USA) was added (100 μL/well), and incubated at room temperature for 5 min. Reactions were stopped with 100 μL/well of stop solution (Surmodics IVD, Inc.), and OD at 450 nm was measured using an ELISA plate reader (EMax Plus Microplate Reader^®^ Molecular Devices, San Jose, CA, USA) operated with a commercial software (Softmax Pro 7.0, Molecular Devices). Serum antibody response was expressed as a sample-to-positive (S/P) ratio with a 0.2 cutoff.

## 3. Results

### 3.1. APPV RNA Detection by RT-qPCR

Nine gilts originating from APPV-positive litters affected by congenital tremors were transported from a commercial farm at approximately six months of age to a research facility for monthly sampling to characterize viral persistence. All serum samples collected after arrival were PCR-negative for APPV. However, individual oral swabs were positive for all the gilts except one (49) throughout the study period (Table 1). In contrast, rectal swabs were only sporadically positive, and three out of nine gilts never tested PCR-positive in rectal swabs. Gilt 49 was the only animal that remained APPV-negative across all sample types during monthly sampling. Monthly group oral fluid samples were positive throughout the study (Supplemental Table S1).

The boar (contact group 1) was tested monthly for approximately three months, and all serum and swab samples were negative for APPV (Table 2). In contrast, both animals in contact group 2, which were sampled weekly, had at least one positive serum or oral swab sample (Table 2). Pig 209 had a positive serum sample starting at 35 days post contact (dpc); however, pig 208 did not have a positive serum or oral swab sample until 69 dpc.

### 3.2. APPV Antibody Detection by ELISA

The timing of antibody response and S/P ratios among the gilts in this study were highly variable (Table 3). Some animals had an early response, with gilts 51 and 58 having S/P ratios above the cutoff on the first sample collected upon arrival in May. In contrast, gilts 59 and 54 did not exceed the cutoff until September and October, respectively. Of note, gilt 49 was the only animal without evidence of an antibody response based on ELISA testing.

Though the boar from contact group 1 did not have any PCR-positive samples, there was evidence of an antibody response after contact with the gilts (Table 3). In contact group 2, only pig 209 had a detectable immune response, with an S/P ratio exceeding the cutoff.

### 3.3. No Detection of APPV in Neonates

On the day of birth (d0), all the piglets were sampled by blood collection and oral swabbing to assess vertical transmission of APPV from sows to their litters. All piglet serum and oral swab samples were PCR-negative on d0. Of note, sows were also oral swabbed on d0 (October, Table 1), and four out of five were PCR-positive, with Ct values ranging from 30.3 to 32.2. The piglets were observed daily for clinical signs of CT, and none demonstrated visible tremors. Approximately four weeks later (d31), a subset of the piglets from each litter was resampled, and all samples remained PCR-negative for APPV. Two piglets per litter were necropsied on d35, and all cerebellum samples were PCR-negative for APPV.

### 3.4. Tissue Distribution of APPV

Gilt 27 was smaller than the other gilts and was euthanized prior to breeding. Only the cerebellum was collected at necropsy. For the remaining animals, a subset of tissues was collected based on previously reported APPV-positive tissues and those potentially contributing to prolonged detection of APPV in oral fluids [25]. In addition, reproductive tissues were collected. All the gilts had PCR-positive cerebellum samples, with Ct values ranging from 20.6 to 35.0 (Table 4). In addition to the cerebellum, three gilts had positive mandibular salivary glands, and four gilts had PCR-positive tonsils and palatoglossal arches. Gilts 26 and 49 were the only animals (of the remaining eight) without additional PCR-positive tissues beyond the cerebellum. None of the gilts had PCR-positive uterus or ovary samples.

The mandibular lymph node and mandibular salivary gland of the boar (contact group 1) were PCR-positive (Table 4). Both animals in contact group 2 had PCR-positive TBLNs, and one (208) had a PCR-positive tonsil. In contrast to the gilts, only one out of the three contact animals (208) had PCR-positive cerebellum tissue. Reproductive tissues were PCR-positive in both the male and female in contact group 2.

## 4. Discussion

The aims of this study were to better characterize the persistence of APPV infection and determine its transmission potential to inform control measures in the swine industry. To accomplish these goals, nine gilts from a longitudinal APPV field study were transported to a research facility at approximately six months of age for further sampling, breeding, and exposure to naïve contact animals [19]. Viremia was not detected in any gilts after arrival; however, the majority had oral swabs that were consistently PCR-positive for APPV. Gilts were able to transmit the virus to contact animals during breeding and gestation but did not transmit the virus to their litters during the first four weeks of life, despite continued PCR-positive oral swabs in some animals. Maternal immunity transferred through colostrum may have contributed to the protection of piglets and prevention of transmission.

This study demonstrated individual differences in shedding patterns and the development of immune responses, which were similarly observed in animals in the previous longitudinal study [19]. One animal (49) did not have any PCR-positive samples during this study and did not develop a measurable immune response; however, this animal had clinical evidence of CT, was APPV-positive at birth, and cerebellum tissue collected at necropsy was PCR-positive. In addition, it was housed with APPV-positive gilts for the duration of the study. Hypotheses for the lack of observed humoral response could be that this animal’s innate immune response or maternal antibodies were able to clear circulating virus prior to the development of an adaptive immune response. Two gilts had a measurable antibody response in the first serum sample collected, whereas others did not have detectable responses until 4–5 months after arrival. Pestiviruses are known to cause immunosuppression in infected animals, which may have contributed in this case to the delayed humoral response observed in animals in this study [25]. The variability in viral dynamics and host response complicates the development of control and prevention strategies. No clinical signs were observed during the study, indicating that animals can actively shed APPV without overt clinical signs and require diagnostic testing for detection.

Persistent infections have been demonstrated in multiple pestiviruses during trans-placental transmission; however, CSFV has also been experimentally demonstrated to induce persistent infection following postnatal exposure [22]. In this study, it remains unclear whether infection occurred transplacentally or postnatally. Consistent with previous APPV studies, prolonged detection of APPV was observed, including sustained PCR-positive oral fluids and detection in tissues at necropsy [15,26,27]. Multiple tissues associated with the oral cavity, including the salivary glands, the tonsil, and the palatoglossal arch, were APPV-positive at necropsy, which could contribute to the extended detection of APPV in oral fluids [28].

Bodily secretions may contribute to transmission in addition to vertical transmission from sows to piglets, which has been demonstrated experimentally [16,29]. Semen and preputial swabs from boars in other studies have tested PCR-positive, suggesting an additional potential route of transmission [15,30,31]. Horizontal transmission was demonstrated in both contact groups in this study. Though PCR-positive antemortem samples were limited, there was evidence of antibody responses and multiple PCR-positive tissues at necropsy. While the boar in contact group 1 did not have PCR-positive reproductive tissues, both animals in contact group 2 had PCR-positive reproductive tissues. Due to the delayed detection of APPV in pig 208 compared to 209, it is not possible to determine whether infection occurred directly from the gilts or via pig 209. This delay may also explain the absence of a detectable antibody response in pig 208.

Tissue PCR results further support consistent localization in the cerebellum, although most reports have focused on neonates with CT [12,28,32,33]. This study demonstrates that APPV can persist in the cerebellum of gilts months after resolution of CT. The cerebellum may serve as an immune-privileged site, allowing viral persistence [26]. Two contact animals did not have detectable APPV in the cerebellum. Timing of infection may influence viral dissemination to central nervous system (CNS) tissues, with neonates exposed in utero or shortly after birth having limited defenses compared to more immunologically mature animals.

The limitations of this study complicate definitive conclusions on the transmission of this virus. Vertical transmission was not demonstrated from gilts to their litters in this study; however, the study was limited by the small sample size. There is still reason to believe sows could transmit to their piglets based off evidence of transmission that was observed between these gilts and animals in the naïve contact groups, as well as continued PCR-positive oral swabs. Maternal antibodies may have contributed to the lack of observed vertical transmission. In addition, due to difficulties in isolating the virus in cell culture, virus isolation was not performed, which would have been able to demonstrate definitively if there was a live replicating virus in both the primary and contact animal samples.

Preventing APPV transmission may be challenging due to the absence of clinical signs in many PCR-positive animals and persistent shedding in some animals. These findings suggest that oral fluids represent a consistent and practical sample type for APPV detection and may be useful for informing animal movement decisions. Future research is needed to better understand individual variability in shedding patterns and immune responses, and whether these are associated with the timing of infection (in utero vs. postnatal).

## Figures and Tables

**Table 1:** PCR results for monthly swabbing of the gilts. Positive samples have Ct values less than or equal to 35 and are bold.

Pig	May		June		July		August		September		October	
	OS	RS	OS	RS	OS	RS	OS	RS	OS	RS	OS	RS
6	NS	**30.6**	**31.0**	**33.2**	**30.2**	**32.6**	**32.1**	Neg	**31.4**	Neg	**31.0**	NS
26	NS	Neg	**34.3**	Neg	**34.2**	Neg	**33.6**	Neg	**33.2**	Neg	Neg	NS
27	NS	**32.1**	**31.2**	**34.1**	**31.7**	Neg	**30.7**	**34.4**	NS	NS	NS	NS
31	NS	Neg	**30.2**	Neg	**31.0**	Neg	**31.6**	Neg	**31.2**	Neg	**32.2**	NS
49	NS	Neg	Neg	Neg	Neg	Neg	Neg	Neg	NS	NS	NS	NS
51	NS	**31.5**	**29.0**	Neg	**31.0**	Neg	**30.7**	Neg	NS	NS	NS	NS
54	NS	**30.5**	**28.2**	**32.0**	**30.0**	Neg	**29.3**	Neg	**30.0**	Neg	**30.3**	NS
58	NS	**29.4**	**30.2**	Neg	**31.0**	Neg	**31.1**	Neg	NS	NS	NS	NS
59	NS	**34.6**	**31.7**	Neg	**31.6**	Neg	**32.9**	Neg	**33.0**	Neg	**30.4**	NS

OS = oral swab, RS = rectal swab, NS = no sample collected, Neg ≥ 35 Ct value, Positive values are bold.

**Table 2:** PCR results for serum and swabs collected from contact animals. Positive samples have Ct values less than or equal to 35 and are bold.

Pig	6799 (Contact Group 1)			208 (Contact Group 2)			209 (Contact Group 2)		
dpc	OS	RS	S	OS	RS	S	OS	RS	S
0	Neg	Neg	Neg	Neg	Neg	Neg	Neg	Neg	Neg
7	NS	NS	NS	Neg	Neg	Neg	Neg	Neg	Neg
14	NS	NS	NS	Neg	Neg	Neg	Neg	Neg	Neg
21	NS	NS	NS	Neg	Neg	Neg	Neg	Neg	Neg
28	Neg	Neg	Neg	Neg	Neg	Neg	Neg	Neg	Neg
35	NS	NS	NS	Neg	Neg	Neg	Neg	Neg	**33.4**
42	NS	NS	NS	Neg	Neg	Neg	**33.2**	Neg	Neg
49	Neg	Neg	Neg	Neg	Neg	Neg	**34.7**	Neg	**32.2**
56	NS	NS	NS	Neg	Neg	Neg	Neg	Neg	Neg
69	NS	NS	NS	**26.2**	Neg	**30.2**	Neg	Neg	Neg

dpc = days post contact, OS = oral swab, RS = rectal swab, S = serum, NS = no sample collected, Neg ≥ 35 Ct value, Positive values are bold.

**Table 3:** APPV E^rns^ ELISA S/P ratios. S/P ratios above the cutoff of 0.2 are bold.

Group	Pig	May	June	July	August	September	October	November
Primary	6	0.115	0.106	0.035	**0.409**	**0.643**	**1.306**	NS
Primary	26	0.202	0.326	**1.436**	**1.900**	**2.362**	**1.997**	NS
Primary	27	0.025	0.029	0.046	**0.453**	NS	NS	NS
Primary	31	0.102	0.146	**0.359**	**0.490**	**0.511**	**0.539**	NS
Primary	49	0.139	0.111	0.057	0.035	0.078	NS	NS
Primary	51	**1.079**	**1.254**	**1.956**	**1.734**	**1.958**	NS	NS
Primary	54	0.027	0.040	0.051	0.053	0.127	**0.435**	NS
Primary	58	**1.523**	**1.288**	**1.886**	**1.766**	**0.638**	NS	NS
Primary	59	0.078	0.052	0.070	0.149	**0.807**	**1.492**	NS
Contact group 1	6799	NS	0.135	NS	NS	**2.760**	NS	NS
Contact group 2	208	NS	NS	NS	NS	−0.006	NS	0.002
Contact group 2	209	NS	NS	NS	NS	−0.008	NS	**1.327**

NS = no sample collected, Positive values are bold.

**Table 4:** Tissue PCR results. Positive samples have Ct values less than or equal to 35 and are bold.

Pig	Uterus	Ovary	Testicle	Cerebellum	TBLN	Mandibular LN	Mandibular SG	Tonsil	PG Arch
6	Neg	Neg	NA	**28.7**	Neg	Neg	**30.9**	**34.7**	Neg
26	Neg	Neg	NA	**30.6**	Neg	Neg	Neg	Neg	Neg
31	Neg	Neg	NA	**25.5**	Neg	Neg	Neg	Neg	**30.2**
49	Neg	NS	NA	**32.0**	Neg	Neg	Neg	Neg	Neg
51	Neg	Neg	NA	**25.8**	Neg	Neg	**25.1**	**30.5**	**28.9**
54	Neg	Neg	NA	**26.3**	Neg	Neg	Neg	**30.0**	**33.2**
58	Neg	Neg	NA	**27.5**	Neg	Neg	Neg	**34.9**	**29.8**
59	Neg	Neg	NA	**30.6**	Neg	Neg	**31.2**	Neg	Neg
27	NS	NS	NA	**20.6**	NS	NS	NS	NS	NS
6799	NA	NA	Neg	Neg	Neg	**33.4**	**32.4**	Neg	Neg
208	NA	NA	**26.2**	**35.0**	**24.4**	NS	Neg	**29.9**	NS
209	**31.2**	**32.1**	NA	Neg	**32.3**	NS	Neg	Neg	NS

TBLN = tracheobronchial lymph node, LN = lymph node, SG = salivary gland, PG = palatoglossal, NA = not applicable, NS = no sample collected, Neg ≥ 35 Ct value, Positive values are bold.

## Data Availability

The original contributions presented in this study are included in the article/Supplementary Materials. Further inquiries can be directed to the corresponding author.

## Supplementary Materials

The following supporting information can be downloaded at: https://www.mdpi.com/article/10.3390/v18060590/s1, Table S1: PCR Ct values of monthly group oral fluids collected from the gilts housed in two pens.

## References

[1] Hause B.M., Collin E.A., Peddireddi L., Yuan F., Chen Z., Hesse R.A., Gauger P.C., Clement T., Fang Y., Anderson G. (2015). Discovery of a novel putative atypical porcine pestivirus in pigs in the USA. J. Gen. Virol..

[2] Kinsley A.T. (1922). Dancing pigs?. Vet. Med..

[3] Pan S., Mou C., Chen Z. (2019). An emerging novel virus: Atypical porcine pestivirus (APPV). Rev. Med. Virol..

[4] Postel A., Meyer D., Cagatay G.N., Feliziani F., De Mia G.M., Fischer N., Grundhoff A., Milicevic V., Deng M.C., Chang C.Y. (2017). High Abundance and Genetic Variability of Atypical Porcine Pestivirus in Pigs from Europe and Asia. Emerg. Infect. Dis..

[5] Munoz-Gonzalez S., Canturri A., Perez-Simo M., Bohorquez J.A., Rosell R., Cabezon O., Segales J., Domingo M., Ganges L. (2017). First report of the novel atypical porcine pestivirus in Spain and a retrospective study. Transbound. Emerg. Dis..

[6] Cagatay G.N., Antos A., Meyer D., Maistrelli C., Keuling O., Becher P., Postel A. (2018). Frequent infection of wild boar with atypical porcine pestivirus (APPV). Transbound. Emerg. Dis..

[7] Choe S., Park G.N., Cha R.M., Hyun B.H., Park B.K., An D.J. (2020). Prevalence and Genetic Diversity of Atypical Porcine Pestivirus (APPV) Detected in South Korean Wild Boars. Viruses.

[8] Denes L., Biksi I., Albert M., Szeredi L., Knapp D.G., Szilasi A., Balint A., Balka G. (2018). Detection and phylogenetic characterization of atypical porcine pestivirus strains in Hungary. Transbound. Emerg. Dis..

[9] Kaufmann C., Stalder H., Sidler X., Renzullo S., Gurtner C., Grahofer A., Schweizer M. (2019). Long-Term Circulation of Atypical Porcine Pestivirus (APPV) within Switzerland. Viruses.

[10] Sozzi E., Salogni C., Lelli D., Barbieri I., Moreno A., Alborali G.L., Lavazza A. (2019). Molecular Survey and Phylogenetic Analysis of Atypical Porcine Pestivirus (APPV) Identified in Swine and Wild Boar from Northern Italy. Viruses.

[11] Shiokawa M., Okuwa-Yamauchi Y., Ohashi-Kawai M., Nagai M., Aoki H. (2025). Full-length sequence determination and isolation of infectious particles of an atypical porcine pestivirus from wild boars in Ishikawa Prefecture, Japan. Infect. Genet. Evol..

[12] Anoyatbekova A., Yuzhakov A. (2024). Isolation and Phylogenetic Analysis of Atypical Porcine Pestivirus Isolates Identified in Russian Swine Herds. Viruses.

[13] Beer M., Wernike K., Drager C., Hoper D., Pohlmann A., Bergermann C., Schroder C., Klinkhammer S., Blome S., Hoffmann B. (2017). High Prevalence of Highly Variable Atypical Porcine Pestiviruses Found in Germany. Transbound. Emerg. Dis..

[14] Mosena A.C.S., Weber M.N., da Cruz R.A.S., Cibulski S.P., da Silva M.S., Puhl D.E., Hammerschmitt M.E., Takeuti K.L., Driemeier D., de Barcellos D. (2018). Presence of atypical porcine pestivirus (APPV) in Brazilian pigs. Transbound. Emerg. Dis..

[15] Schwarz L., Riedel C., Hogler S., Sinn L.J., Voglmayr T., Wochtl B., Dinhopl N., Rebel-Bauder B., Weissenbock H., Ladinig A. (2017). Congenital infection with atypical porcine pestivirus (APPV) is associated with disease and viral persistence. Vet. Res..

[16] de Groof A., Deijs M., Guelen L., van Grinsven L., van Os-Galdos L., Vogels W., Derks C., Cruijsen T., Geurts V., Vrijenhoek M. (2016). Atypical Porcine Pestivirus: A Possible Cause of Congenital Tremor Type A-II in Newborn Piglets. Viruses.

[17] Cagatay G.N., Meyer D., Wendt M., Becher P., Postel A. (2019). Characterization of the Humoral Immune Response Induced after Infection with Atypical Porcine Pestivirus (APPV). Viruses.

[18] Folgueiras-Gonzalez A., van den Braak R., Simmelink B., Deijs M., van der Hoek L., de Groof A. (2020). Atypical Porcine Pestivirus Circulation and Molecular Evolution within an Affected Swine Herd. Viruses.

[19] Buckley A.C., Mora-Diaz J.C., Magtoto R.L., Hulzen A.V., Ferreyra F.M., Falkenberg S.M., Gimenez-Lirola L.G., Arruda B.L. (2023). Dynamics of Infection of Atypical Porcine Pestivirus in Commercial Pigs from Birth to Market: A Longitudinal Study. Viruses.

[20] Schweizer M., Peterhans E. (2014). Pestiviruses. Annu. Rev. Anim. Biosci..

[21] Peterhans E., Schweizer M. (2013). BVDV: A pestivirus inducing tolerance of the innate immune response. Biologicals.

[22] Munoz-Gonzalez S., Ruggli N., Rosell R., Perez L.J., Frias-Leuporeau M.T., Fraile L., Montoya M., Cordoba L., Domingo M., Ehrensperger F. (2015). Postnatal persistent infection with classical Swine Fever virus and its immunological implications. PLoS ONE.

[23] Prickett J.R., Zimmerman J.J. (2010). The development of oral fluid-based diagnostics and applications in veterinary medicine. Anim. Health Res. Rev..

[24] Arruda B.L., Falkenberg S., Mora-Diaz J.C., Matias Ferreyra F.S., Magtoto R., Gimenez-Lirola L. (2022). Development and Evaluation of Antigen-Specific Dual Matrix Pestivirus K ELISAs Using Longitudinal Known Infectious Status Samples. J. Clin. Microbiol..

[25] Tarradas J., de la Torre M.E., Rosell R., Perez L.J., Pujols J., Munoz M., Munoz I., Munoz S., Abad X., Domingo M. (2014). The impact of CSFV on the immune response to control infection. Virus Res..

[26] Buckley A.C., Falkenberg S.M., Palmer M.V., Arruda P.H., Magstadt D.R., Schwartz K.J., Gatto I.R., Neill J.D., Arruda B.L. (2021). Distribution and persistence of atypical porcine pestivirus in experimentally inoculated pigs. J. Vet. Diagn. Investig..

[27] Bergfeldt A., Myrmel M., Ranheim B., Aae F., Sorby R. (2025). Cerebellar hypomyelination, white matter vacuolization, and prolonged presence of atypical porcine pestivirus in pigs with congenital tremor type A-II. Vet. Pathol..

[28] Liu J., Li Z., Ren X., Li H., Lu R., Zhang Y., Ning Z. (2019). Viral load and histological distribution of atypical porcine pestivirus in different tissues of naturally infected piglets. Arch. Virol..

[29] Arruda B.L., Arruda P.H., Magstadt D.R., Schwartz K.J., Dohlman T., Schleining J.A., Patterson A.R., Visek C.A., Victoria J.G. (2016). Identification of a Divergent Lineage Porcine Pestivirus in Nursing Piglets with Congenital Tremors and Reproduction of Disease following Experimental Inoculation. PLoS ONE.

[30] Gatto I.R.H., Arruda P.H., Visek C.A., Victoria J.G., Patterson A.R., Krull A.C., Schwartz K.J., de Oliveira L.G., Arruda B.L. (2018). Detection of atypical porcine pestivirus in semen from commercial boar studs in the United States. Transbound. Emerg. Dis..

[31] Houston G.E., Jones C.K., Woodworth J.C., Palinski R., Paulk C.B., Petznick T., Gebhardt J.T. (2022). Detection and investigation of atypical porcine pestivirus in a swine production system. Front. Vet. Sci..

[32] Postel A., Hansmann F., Baechlein C., Fischer N., Alawi M., Grundhoff A., Derking S., Tenhundfeld J., Pfankuche V.M., Herder V. (2016). Presence of atypical porcine pestivirus (APPV) genomes in newborn piglets correlates with congenital tremor. Sci. Rep..

[33] Gatto I.R.H., Harmon K., Bradner L., Silva P., Linhares D.C.L., Arruda P.H., de Oliveira L.G., Arruda B.L. (2018). Detection of atypical porcine pestivirus in Brazil in the central nervous system of suckling piglets with congenital tremor. Transbound. Emerg. Dis..

