# Supplementary material for: Transmission Dynamics of Gilts Persistently Infected with Atypical Porcine Pestivirus
Source: Viruses. 2026 May 23;18(6):590. doi: 10.3390/v18060590 (PMC13308041; doi:10.3390/v18060590)
Supplement: Supplementary file 1 [file viruses-18-00590-s001.zip › viruses-4277797-supplementary.pdf]

Table S1: PCR Ct values of monthly group oral fluids collected from the gilts housed in two pens.

Pen 2	May	25.01097
Pen 4	May	24.04053
Pen 2	June	25.05778
Pen 4	June	23.31533
Pen 2	July	NS
Pen 4	July	26.18937
Pen 2	August	26.8464
Pen 4	August	23.0769
Pen 2	September	NS
Pen 4	September	NS
Pen 2	October	28.64156
Pen 4	October	27.72477

NS = not sampled
